# New species of *Pseudosperma* (Agaricales, Inocybaceae) from Pakistan revealed by morphology and multi-locus phylogenetic reconstruction

**DOI:** 10.3897/mycokeys.69.33563

**Published:** 2020-07-10

**Authors:** Malka Saba, Danny Haelewaters, Donald H. Pfister, Abdul Nasir Khalid

**Affiliations:** 1 Department of Plant Sciences, Quaid-i-Azam University, Islamabad, 45320, Pakistan Quaid-i-Azam University Islamabad Pakistan; 2 Farlow Herbarium of Cryptogamic Botany, Harvard University, Cambridge, Massachusetts, USA Harvard University Cambridge United States of America; 3 Department of Botany and Plant Pathology, Purdue University, West Lafayette, Indiana, USA Purdue University West Lafayette United States of America; 4 Faculty of Science, University of South Bohemia, České Budějovice, Czech Republic University of South Bohemia České Budějovice Czech Republic; 5 Department of Botany, University of the Punjab, Lahore, Pakistan University of the Punjab Lahore Pakistan

**Keywords:** Ectomycorrhizal fungi, molecular systematics, phylogeny, *Pinusroxburghii*, southern Asia, taxonomy

## Abstract

During fungal surveys between 2012 and 2014 in pine-dominated forests of the western Himalayas in Pakistan, several collections of *Pseudosperma* (Agaricales, Inocybaceae) were made. These were documented, based on morphological and molecular data. During this work, three new species came to light, which are here formally described as *Pseudospermabrunneoumbonatum*, *P.pinophilum* and *P.triacicularis*. These species belong in the genus *Pseudospermafide*Matheny et al. (2019) = Pseudosperma clade *fide*Matheny (2005) = *Inocybe* sect. Rimosaes.s.fideLarsson et al. (2009). Macro- and micro-morphological descriptions, illustrations and molecular phylogenetic reconstructions of the studied taxa are provided. The new species are differentiated from their close relatives by basidiospore size and colouration of basidiomata. Molecular phylogenetic relationships are inferred using ITS (ITS1–5.8S–ITS2), nrLSU and mtSSU sequence data. All three newly-described taxa likely share an ectomycorrhizal association with trees in the genus *Pinus*. In addition, five names are recombined in *Inosperma*, *Mallocybe* and *Pseudosperma*. These are *Inospermavinaceobrunneum*, *Mallocybeerratum*, *Pseudospermaalboflavellum*, *Pseudospermafriabile* and *Pseudospermaneglectum*.

## Introduction

*Inocybe* (Fr.) Fr. (Agaricales, Inocybaceae) in the broad sense (sensu lato) is a highly diverse, ectomycorrhizal genus comprising about 735 known species worldwide (Ullah et al. 2018). *Inocybe* has a widespread distribution and is found commonly in temperate areas and, to a lesser extent, in the tropics (Matheny et al. 2009, Bougher et al. 2012, Matheny et al. 2012). Multi-locus phylogenies of the Inocybaceae by Matheny et al. (2002, 2009) and Matheny (2005) have confirmed that the family is monophyletic. Matheny (2005, 2009) recognised seven major clades within the Inocybaceae; clade names were given with a suggestion to recognise each informally at the generic rank within the family.

InocybesectionRimosae sensu stricto (*fide*Larsson et al. 2009, = clade Pseudosperma*fide*Matheny 2005), traditionally placed in subgenusInosperma (Kuyper 1986, Kobayashi 2002), is one of the seven major clades in the Inocybaceae. Species of this clade are typically characterised by a rimose pileus surface; furfuraceous to furfuraceous-fibrillose stipe; absence of metuloids and pleurocystidia; smooth, elliptical to indistinctly phaseoliform basidiospores; and cylindrical to clavate cheilocystidia. Unlike species in clades Mallocybe and Inosperma (*fide*Matheny 2005) and the genera *Auritella* Matheny & Bougher and *Tubariomyces* Esteve-Rav. & Matheny, all of which also lack pleurocystidia, the basidia of species in the Pseudosperma clade are hyaline and not necropigmented. The Nothocybe clade is represented by only one species, *I.distincta* K.P.D. Latha & Manim. This species also lacks pleurocystidia and can be differentiated based on molecular phylogenetic data (Latha et al. 2016). Some lineages in the Pseudosperma clade are composed of multiple cryptic species (Ryberg et al. 2008) and they form ectomycorrhizal associations with a broad range of host trees, both gymnosperms and angiosperms (Kuyper 1986, Stangl 1989, Jacobsson 2008).

Based on a six-locus phylogeny of the family *Inocybaceae*, Matheny et al. (2019) formally proposed genus names for the different clades: *Inocybe* sensu stricto, *Inosperma* (Kühner) Matheny & Esteve-Rav. (elevated from subgenus-level), *Mallocybe* (Kuyper) Matheny, Vizzini & Esteve-Rav. (elevated from subgenus-level), *Nothocybe* Matheny & K.P.D. Latha and *Pseudosperma* Matheny & Esteve-Rav., in addition to *Auritella* and *Tubariomyces* that were previously described. The authors decided to provide a formal generic system to name the different clades, because this allows better communication and provides the taxonomic precision needed for conservation issues and identification of biodiversity hot spots.

During an investigation of ectomycorrhizal fungi associated with pine species in Pakistan, three species of Pseudospermawith affiliation tosect.Rimosae s.s. were collected in the vicinity of pure stands of *Pinusroxburghii* Sarg. and *P.wallichiana* A.B. Jacks. The species were documented, based on morphological and molecular phylogenetic data. In this paper, we describe these taxa as new species, *P.brunneoumbonatum*, *P.pinophilum* and *P.triaciculare*. This is the first study in which a combination of morphological and multi-locus phylogenetic data was used to describe species of Inocybe sensu lato in sect. Rimosae s.s. – now genus *Pseudosperma* – from Pakistan.

## Material and methods

### Morphological studies

Basidiomata were collected, described and photographed in the field. Colours were compared to the Munsell Soil Color Charts (1975) guide. Collections were dried using a food dehydrator (at 39 °C for 7–9 hours). Microscopic characters were observed in the laboratory using hand-cut sections of basidiomata mounted in a 5% aqueous solution of potassium hydroxide (KOH) and in Congo red. Micromorphological analysis, photographs and measurements were made, using an Olympus BX40 light microscope with Olympus XC50 digital camera and Microsuite special edition software 3.1 (Soft imaging solutions GmbH). Thirty basidiospores were measured from each collection cited. Measurements include the range with extremes provided in parentheses. Q values (length/width ratios) and mean values (average basidiospore length and width) are also provided. Line drawings were made with a Leitz camera Lucida (Wetzlar, Germany). Collections of the newly-described species are deposited at LAH (University of the Punjab Herbarium, Lahore) and FH (Farlow Herbarium, Harvard University).

### DNA extraction, PCR amplification and DNA sequencing

Genomic DNA was extracted from a 20 mg piece of dried tissue by a modified CTAB method (Lee et al. 1988). Loci examined during this study include the complete ITS region (ITS1–5.8S–ITS2) of the nuclear ribosomal RNA gene (hereafter ITS), the first ca. 900 bp of the nuclear 28S rRNA gene (nrLSU) and the mitochondrial small subunit rRNA gene (mtSSU).

Primers used for amplification were: ITS1F (Gardes and Bruns 1993) and ITS4 (White et al. 1990) for ITS; LR0R and LR5 for nrLSU (Vilgalys and Hester 1990); and MS1 and MS2 for mtSSU (White et al. 1990). The amplification reaction mixture contained 2.5 µl Econo buffer, 0.5 µl dNTPs, 1.25 µl each primer, 0.125 µl Econo Taq, 14.375 µl of deionised water and 5 µl of template DNA. Thermal profile of PCR for ITS was initial denaturation at 94 °C for 1 min.; then 35 cycles of denaturation at 94 °C for 1 min, annealing at 53 °C for 1 min and extension at 72 °C for 1 min; and final extension at 72 °C for 8 min. For nrLSU: 94 °C for 2 min; then 40 cycles of 94 °C for 1 min, 52 °C for 1 min and 72 °C for 1:30 min; and 72 °C for 5 min. For mtSSU: 95 °C for 10 min; then 30 cycles of 95 °C for 30 sec, 52 °C for 30 sec and 72 °C for 40 sec; and 72 °C for 7 min.

PCR products were run on 1% agarose gel, stained with ethidium bromide and bands were visualised under a UV transilluminator. Amplified PCR products of the ITS region were sent for purification and bidirectional sequencing to Macrogen (Republic of Korea). PCR products of 28S and 16S were purified using QIAquick PCR purification kit (Qiagen, Stanford, California) as per manufacturer’s guidelines and sequencing reactions were performed using the Big Dye Terminator v3.1 Cycle Kit (Life Technologies, Carlsbad, California). Sequencing was carried out using the same primers as those used for PCR.

### Sequence alignment and phylogenetic analysis

Sequences were manually edited and assembled in BioEdit v7.2.6 (Hall 1999). Generated ITS sequences were trimmed with the conserved motifs 5’–CATTA– and –GACCT–3’ (Dentinger et al. 2011) and the alignment portion between these motifs was included in subsequent analyses. BLASTn searches were performed in NCBI GenBank. Three data matrices for phylogenetic inferences were prepared: a concatenated ITS–nrLSU–mtSSU dataset of *Rimosae* s.s. and Inosperma clades (dataset #1); a concatenated ITS–nrLSU–mtSSU dataset of *Rimosae* s.s. subclade A (dataset #2); and an extended nrLSU dataset of *Rimosae* s.s. subclade A (dataset #3). We applied the clade names used by Larsson et al. (2009) in the methods and results sections to maintain consistency and clarity.

Sequences were downloaded from NCBI GenBank (https://www.ncbi.nlm.nih.gov/genbank/). The majority of sequences were generated in the studies of Larsson et al. (2009) and Ryberg et al. (2008), complemented by nrLSU sequences from more recent papers and our newly-generated sequences (details and references in Table 1). Sequences were aligned by locus (ITS+nrLSU, mtSSU) using Muscle v3.7 (Edgar 2004), available in the Cipres Science Gateway (Miller et al. 2010). Ambiguously-aligned regions were detected and removed using trimAl v1.3 (Capella-Gutiérrez et al. 2009), with the following parameters: 60% gap threshold, 50% minimal coverage. The ITS1, 5.8S, ITS2 and nrLSU loci were extracted from the aligned ITS+nrLSU dataset. This allowed us to select substitution models for each region, which is important because there are different rates of evolution within and amongst these components and rDNA loci (e.g. Hillis and Dixon 1991, discussion in Haelewaters et al. 2018).

**Table 1.** Isolates used in phylogenetic analyses, with geographic origin and GenBank accession numbers. Accession numbers of sequences generated during this study are in boldface. Explanation of datasets: #1 = concatenated ITS–nrLSU–mtSSU dataset of *Rimosae* s.s. and Inosperma clades, #2 = concatenated ITS–nrLSU–mtSSU dataset of *Rimosae* s.s. subclade A, #3 = extended nrLSU dataset of *Rimosae* s.s. subclade A (dataset #3). X under #1, #2, #3 = sequence(s) were used in the respective dataset. OUT = outgroup.

Species	Isolate	Geographic origin	GenBank		Reference(s)	Dataset		
			ITS/nrLSU	mtSSU		#1	#2	#3
*Alnicolabohemica*	EL71b-03	Sweden	FJ904179	FJ904243	Larsson et al. (2009)	OUT		OUT
*Alnicolasalicis*	EL71a-03	Sweden	FJ904180		Larsson et al. (2009)	OUT		OUT
*Alnicolasubmelinoides*	TAA185174	Estonia	AM882885		Ryberg et al. (2008)	OUT		OUT
*Conocybesiliginea*	LÖ93-04	Sweden	DQ389731		Larsson and Orstadius (2008)	OUT		
*Crepidotuscalolepis*	EL14-08	Sweden	FJ904178	FJ904242	Larsson et al. (2009)	X		
*Crepidotusmollis*	EL45-04	Sweden	AM882996		Ryberg et al. (2008)	X		
*Inospermaadaequatum*	PC2008-0014	Great Britain	FJ904177	FJ904240	Larsson et al. (2009)	X		
*Inospermaadaequatum*	MR00022	Sweden	AM882706	FJ904241	Ryberg et al. (2008), Larsson et al. (2009)	X		
*Inospermabongardii*	EL123-04	Sweden	AM882941	FJ904186	Ryberg et al. (2008), Larsson et al. (2009)	X		
Inospermacf.calamistrata	KHL13071	Costa Rica	AM882948		Ryberg et al. (2008)	X		
*Inospermacervicolor*	SJ04024	Sweden	AM882939	FJ904185	Ryberg et al. (2008), Larsson et al. (2009)	X		
*Inospermacookei*	MR00035	Sweden	AM882954		Ryberg et al. (2008)	X		
*Inospermacookei*	EL191-06	Great Britain	FJ904173	FJ904234	Larsson et al. (2009)	X		
*Inospermacookei*	EL70a-03	Sweden	AM882953		Ryberg et al. (2008)	X		
*Inospermacookei*	EL73-05	Sweden	AM882955		Ryberg et al. (2008)	X		
*Inospermacookei*	EL109-04	Sweden	AM882956	FJ904233	Ryberg et al. (2008), Larsson et al. (2009)	X		
Inospermacf.cookei	EL104-04	Sweden	AM882952		Ryberg et al. (2008)	X		
*Inospermaerubescens*	TAA185164	Estonia	AM882950		Ryberg et al. (2008)	X		
*Inospermaerubescens*	KGN980714	Sweden	AM882951	FJ904239	Ryberg et al. (2008), Larsson et al. (2009)	X		
*Inospermaerubescens*	BH910707	Sweden	AM882949		Ryberg et al. (2008)	X		
*Inospermamaculatum*	EL74-05	Sweden	AM882959		Ryberg et al. (2008)	X		
*Inospermafulvum*	EL78-03	Sweden	AM882962		Ryberg et al. (2008)	X		
*Inospermafulvum*	EL166-08	Sweden	FJ904171	FJ904231	Larsson et al. (2009)	X		
*Inospermafulvum*	EL114-06	Sweden	FJ904170		Larsson et al. (2009)	X		
*Inospermafulvum*	SJ05029	Sweden	AM882994	FJ904230	Ryberg et al. (2008), Larsson et al. (2009)	X		
*Inospermafulvum*	EL247-06	France	FJ904169		Larsson et al. (2009)	X		
*Inospermafulvum*	PAM01100120	France	FJ904168		Larsson et al. (2009)	X		
*Inospermafulvum*	SJ06007	Sweden	FJ904167		Larsson et al. (2009)	X		
*Inospermamaculatum*	MR00020	Sweden	AM882958		Ryberg et al. (2008)	X		
*Inospermamaculatum*	EL121-04	Sweden	AM882957	FJ904232	Ryberg et al. (2008), Larsson et al. (2009)	X		
*Inospermamaculatum*	EL58-03	Sweden	AM882963		Ryberg et al. (2008)	X		
*Inospermamaculatum*	EL126-04	Sweden	AM882964		Ryberg et al. (2008)	X		
*Inospermamaculatum*	EL182-08	Slovenia	FJ904172		Larsson et al. (2009)	X		
*Inospermaquietiodor*	RP980718	Sweden	FJ936169	FJ904238	Larsson et al. (2009)	X		
*Inospermaquietiodor*	LAS97-067	Sweden	AM882974		Ryberg et al. (2008)	X		
*Inospermaquietiodor*	LAS94-023	Sweden	AM882961		Ryberg et al. (2008)	X		
*Inospermaquietiodor*	PAM01091310	France	FJ936168	FJ904237	Larsson et al. (2009)	X		
*Inospermaquietiodor*	EL115-04	Sweden	AM882960	FJ904236	Ryberg et al. (2008), Larsson et al. (2009)	X		
*Inospermaquietiodor*	JV20202	Norway	FJ904174	FJ904235	Larsson et al. (2009)	X		
*Inospermarhodiolum*	PAM00090117	France	FJ904176		Larsson et al. (2009)	X		
*Inospermarhodiolum*	EL223-06	France	FJ904175		Larsson et al. (2009)	X		
*Inospermasubhirsutum*	EL45-05	Norway		FJ904187	Larsson et al. (2009)	X		
*Inospermavirosum*	TBGT753	India	KT329458		Pradeep et al. 2016			X
*Inospermavirosum*	CAL1383	India	KY549138		K.P. Deepna Latha and P. Manihoman unpubl.			X
*Mallocybeagardhii*	EL88-04	Sweden	FJ904123	FJ904182	Larsson et al. (2009)	X		
*Mallocybedulcamara*	EL89-06	Sweden	FJ904122	FJ904181	Larsson et al. (2009)	X		
*Mallocybefulvipes*	EL37-05	Norway	AM882858	FJ904184	Ryberg et al. (2008), Larsson et al. (2009)	X		
*Mallocybeterrigena*	EL117-04	Sweden	AM882864	FJ904183	Ryberg et al. (2008), Larsson et al. (2009)	X		
*Pseudospermaaestivum*	BK18089706	USA, Utah	EU600847		Matheny et al. (2009)		X	X
*Pseudospermaalboflavellum*	TBGT11280	India	KP171058		Pradeep et al. (2016)			X
*Pseudospermaarenicola*	RC GB99-014	France	FJ904134	FJ904189	Larsson et al. (2009)	X		
*Pseudospermaarenicola*	EL238-06	France	FJ904133	FJ904188	Larsson et al. (2009)	X		
*Pseudospermabreviterincarnatum*	BK18089724	USA, Utah	EU555449		Matheny et al. (2009)			X
*Pseudospermabreviterincarnatum*	BK28080407	USA, Utah	EU555451		Matheny et al. (2009)			X
*Pseudospermabreviterincarnatum*	PBM1914	USA, Washington	JQ319677		Kropp et al. (2013)			X
***Pseudospermabrunneoumbonatum***	**MSM#0053**	**Pakistan**	**MG742419/MG742420**	**n/a**	**This study**	**X**	**X**	**X**
***Pseudospermabrunneoumbonatum***	**MSM#00545**	**Pakistan**	**MG742421/MG742422**	**n/a**	**This study**	**X**	**X**	**X**
*Pseudospermabulbosissimum*	EL51-05	Norway	AM882764		Ryberg et al. (2008)	X	X	X
*Pseudospermabulbosissimum*	EL66-05	Norway	AM882765	FJ904224	Ryberg et al. (2008), Larsson et al. (2009)	X	X	X
*Pseudospermabulbosissimum*	EL37-06	Sweden	FJ904161	FJ904223	Larsson et al. (2009)	X	X	X
*Pseudospermabulbosissimum*	EL75-07	Sweden	FJ904160	FJ904222	Larsson et al. (2009)	X	X	X
*Pseudospermabulbosissimum*	EL88-06	Sweden	FJ904159	FJ904221	Larsson et al. (2009)	X	X	X
*Pseudospermabulbosissimum*	EL30-06	Sweden	FJ904158	FJ904220	Larsson et al. (2009)	X	X	X
*Pseudospermacercocarpi*	BK20069806	USA, Utah	EU600890		Matheny et al. (2009)			X
*Pseudospermacercocarpi*	BK20069807	USA, Utah	JQ319683		Kropp et al. (2013)			X
*Pseudospermadulcamaroides*	EL29-08	USA, Montana	FJ904127		Larsson et al. (2009)	X		
*Pseudospermadulcamaroides*	EL112-06	Sweden	FJ904126	FJ904194	Larsson et al. (2009)	X		
*Pseudospermaflavellum*	EL56-08	Sweden	FJ904131	FJ904198	Larsson et al. (2009)	X		
*Pseudospermaflavellum*	EL137-05	Sweden	AM882776	FJ904199	Ryberg et al. (2008), Larsson et al. (2009)	X		
*Pseudospermaflavellum*	LAS89-030	Sweden	AM882775		Ryberg et al. (2008)	X		
Pseudospermacf.flavellum	GK080924	Great Britain	FJ904129	FJ904196	Larsson et al. (2009)	X		
Pseudospermacf.flavellum	PAM05062502	France	FJ904128	FJ904195	Larsson et al. (2009)	X		
Pseudospermacf.flavellum	EL118-05	Finland	AM882782		Ryberg et al. (2008)	X		
Pseudospermacf.flavellum	BJ920829	Sweden	AM882774		Ryberg et al. (2008)	X		
Pseudospermacf.flavellum	EL90-04	Sweden	AM882773		Ryberg et al. (2008)	X		
*Pseudospermagriseorubidum*	CAL1253	India	KT180327		Deepna Latha and Manimohan (2015)			X
*Pseudospermahygrophorus*	EL97-06	Sweden	FJ904137	FJ904202	Larsson et al. (2009)	X		
*Pseudospermakeralense*	TBGT12854	India	KP171059		Pradeep et al. (2016)			X
*Pseudospermakeralense*	TBGT12828	India	KP171060		Pradeep et al. (2016)			X
*Pseudospermamelliolens*	PAM05052303	France	FJ904148	FJ904211	Larsson et al. (2009)	X	X	X
*Pseudospermamelliolens*	EL224-06	France	FJ904149		Larsson et al. (2009)	X	X	X
Pseudospermacf.microfastigiatum	EL113-06	Sweden	FJ904156	FJ904217	Larsson et al. (2009)	X	X	X
*Pseudospermamimicum*	EBJ961997	Sweden	FJ904124	FJ904191	Larsson et al. (2009)	X		
*Pseudospermamimicum*	TK2004-114	Sweden	AM882781		Ryberg et al. (2008)	X		
*Pseudospermaniveivelatum*	BK21089714	USA, Utah	JQ319695		Kropp et al. (2013)		X	X
*Pseudospermaniveivelatum*	BK27089718	USA, Utah	EU600831		Matheny et al. (2009)		X	X
*Pseudospermaniveivelatum*	Stz12816	USA, Washington	JQ319696		Kropp et al. (2013)		X	X
*Pseudospermaobsoletum*	EL17-04	Sweden	AM882769	FJ904204	Ryberg et al. (2008), Larsson et al. (2009)	X	OUT	X
*Pseudospermaobsoletum*	BJ890915	Sweden	AM882770		Ryberg et al. (2008)	X	OUT	X
*Pseudospermaoccidentale*	PBM525	USA, Washington	AY038321		Matheny et al. (2002)			X
*Pseudospermaoccidentale*	BK27089703	USA, Utah	EU600893		Matheny et al. (2009)			X
*Pseudospermapakistanense*	LAH35285	Pakistan	MG958608		Ullah et al. (2018)			X
*Pseudospermapakistanense*	LAH35283	Pakistan	MG958609		Ullah et al. (2018)			X
*Pseudospermaperlatum*	BJ940922	Sweden	AM882772		Ryberg et al. (2008)	X	OUT	X
*Pseudospermaperlatum*	EL74-04	Sweden	AM882771	FJ904205	Ryberg et al. (2008), Larsson et al. (2009)	X	OUT	X
***Pseudospermapinophilum***	**MSM#0046**	**Pakistan**	**MG742414/MG742418**	**MG742416**	**This study**	**X**	**X**	**X**
***Pseudospermapinophilum***	**MSM#0047**	**Pakistan**	**MG742417/MG742415**	**MK474612**	**This study**	**X**	**X**	**X**
*Pseudospermarimosum*	AO2008-0250	Great Britain	FJ904147	FJ904210	Larsson et al. (2009)	X	X	X
*Pseudospermarimosum*	EL118-08	Sweden	FJ904146	FJ904209	Larsson et al. (2009)	X	X	X
*Pseudospermarimosum*	EL102-04	Sweden	AM882761		Ryberg et al. (2008)	X	X	X
*Pseudospermarimosum*	EL211-06	France	FJ904145		Larsson et al. (2009)	X	X	X
*Pseudospermarimosum*	TK97-156	Sweden	AM882844		Ryberg et al. (2008)	X	X	
*Pseudospermarimosum*	PAM03110904	France	FJ904144	FJ904208	Larsson et al. (2009)	X	X	X
*Pseudospermarimosum*	EL75-05	Sweden	AM882762	FJ904207	Ryberg et al. (2008), Larsson et al. (2009)	X	X	X
*Pseudospermarimosum*	SJ04007	Sweden	AM882763		Ryberg et al. (2008)	X	X	X
*Pseudospermarimosum*	PAM06112703	Corsica	FJ904143	FJ904206	Larsson et al. (2009)	X	X	X
Pseudospermacf.rimosum	EL71-04	Sweden	AM882786	FJ904193	Ryberg et al. (2008), Larsson et al. (2009)	X		
Pseudospermacf.rimosum	JD2008-0241	Great Britain	FJ904125	FJ904192	Larsson et al. (2009)	X		
Pseudospermacf.rimosum	I116-06	Australia	FJ904142		Larsson et al. (2009)	X		
Pseudospermacf.rimosum	PAM05061101	France	FJ904155	FJ904216	Larsson et al. (2009)	X	X	X
Pseudospermacf.rimosum	JV26578	Estonia	FJ904154	FJ904215	Larsson et al. (2009)	X	X	X
Pseudospermacf.rimosum	EL127-04	Sweden	AM882768	FJ904219	Ryberg et al. (2008), Larsson et al. (2009)	X	X	X
Pseudospermacf.rimosum	TAA185135	Estonia	AM882766		Ryberg et al. (2008)	X	X	X
Pseudospermacf.rimosum	JV22619	Estonia	FJ904157	FJ904218	Larsson et al. (2009)	X	X	X
Pseudospermacf.rimosum	PC080925	Great Britain	FJ904153		Larsson et al. (2009)	X	X	X
Pseudospermacf.rimosum	JV8125	Finland	FJ904152	FJ904214	Larsson et al. (2009)	X	X	X
Pseudospermacf.rimosum	EL81-06	Sweden	FJ904135	FJ904190	Larsson et al. (2009)	X		
*Pseudospermasororium*	Kuoljok0512	Sweden	FJ904150	FJ904212	Larsson et al. (2009)	X	X	X
*Pseudospermasororium*	JV15200	Sweden	FJ904151	FJ904213	Larsson et al. (2009)	X	X	X
*Pseudosperma* sp.	TR138_05	Papua New Guinea	JN975009		Ryberg and Matheny (2012)	X	X	X
*Pseudosperma* sp.	TR133_05	Papua New Guinea	JQ319709		Kropp et al. (2013)	X	X	X
*Pseudosperma* sp.	TR104_05	Papua New Guinea	JN975011		Ryberg and Matheny (2012)	X	X	X
*Pseudospermasquamatum*	SJ08003	Sweden	FJ904136	FJ904201	Larsson et al. (2009)	X		
*Pseudospermasquamatum*	TK96-109	Sweden	AM882780		Ryberg et al. (2008)	x		
*Pseudospermasquamatum*	SJ85048	Norway	AM882778		Ryberg et al. (2008)	X		
*Pseudospermasquamatum*	PAM05052301	France	FJ904132	FJ904200	Larsson et al. (2009)	X		
Pseudospermacf.squamatum	I93-04	Australia	FJ904141		Larsson et al. (2009)	X		
Pseudospermacf.squamatum	I113-05	Australia	FJ904140		Larsson et al. (2009)	X		
Pseudospermacf.squamatum	SJ92-010	Sweden	AM882785		Ryberg et al. (2008)	X		
Pseudospermacf.squamatum	SM92-013	Sweden	AM882783		Ryberg et al. (2008)	X		
Pseudospermacf.squamatum	SJ92-017	Sweden	AM882784		Ryberg et al. (2008)	X		
Pseudospermacf.squamatum	Stordal18318	Norway	FJ904139		Larsson et al. (2009)	X		
Pseudospermacf.squamatum	JV2609	Finland	FJ904138	FJ904203	Larsson et al. (2009)	X		
***Pseudospermatriaciculare***	**MSM#0039**	**Pakistan**	**MG742423/MG742424**	**MG742425**	**This study**	**X**	**X**	**X**
***Pseudospermatriaciculare***	**MSM#0041**	**Pakistan**	**MG742429/MG742430**	**MG742431**	**This study**	**X**	**X**	**X**
***Pseudospermatriaciculare***	**MSM#0040**	**Pakistan**	**MG742426/MG742427**	**MG742428**	**This study**	**X**	**X**	**X**
*Pseudospermaumbrinellum*	JV13699	Finland	FJ904165	FJ904228	Larsson et al. (2009)	X	X	X
*Pseudospermaumbrinellum*	JV17954	Estonia	FJ904166	FJ904229	Larsson et al. (2009)	X	X	X
*Pseudospermaumbrinellum*	PC081010	Great Britain	FJ904164	FJ904227	Larsson et al. (2009)	X	X	X
*Pseudospermaumbrinellum*	PC080816	Great Britain	FJ904163	FJ904226	Larsson et al. (2009)	X	X	X
*Pseudospermaumbrinellum*	PAM01102912	France	FJ904162	FJ904225	Larsson et al. (2009)	X	X	X
*Pseudospermaxanthocephalum*	PAM00100606	France	FJ904130	FJ904197	Larsson et al. (2009)	X		

The data for each locus were concatenated in MEGA7 (Kumar et al. 2016) to create matrices of 2537 bp with sequence data for 123 isolates in the *Rimosae* s.s. and Inosperma dataset (#1); and of 2561 bp for 50 isolates in the *Rimosae* s.s. subclade A dataset (#2). The nrLSU dataset (#3) consisted of 1383 bp for 62 isolates belonging to *Rimosae* s.s. subclade A. Alignments generated during this study are available for download in NEXUS format from the figshare online repository (https://doi.org/10.6084/m9.figshare.c.4701338). Nucleotide substitution models were selected for each locus (ITS1, 5.8S, ITS2, nrLSU, mtSSU) using jModelTest2 (Darriba et al. 2012) by considering the Akaike Information Criterion (AIC). For both concatenated datasets #1 and #2, models were selected for ITS1, 5.8S, ITS2, nrLSU and mtSSU; for dataset #3, the best model was selected for nrLSU. Maximum likelihood was inferred for each dataset under partitioned models using IQ-tree (Nguyen et al. 2015, Chernomor et al. 2016). Ultrafast bootstrapping was done with 1000 replicates (Hoang et al. 2017).

## Results

### Nucleotide alignment datasets and phylogenetic inferences

Concatenated dataset #1 consisted of 2537 characters, of which 1448 were constant and 841 were parsimony-informative. A total of 123 isolates were included, of which *Naucoriabohemica* Velen., *N.salicis* P.D. Orton and *N.submelinoides* (Kühner) Maire (Agaricales, Hymenogastraceae) served as outgroup taxa. The following models were selected by jModelTest2 (AIC): TIM2+I+G (ITS1, -lnL = 6194.8143), TPM2+I (5.8S, -lnL = 445.7026), GTR+G (ITS2, -lnL = 4445.9240), TIM3+I+G (nrLSU, -lnL = 10227.1599) and TVM+I+G (mtSSU, -lnL = 4034.3342). Concatenated dataset #2 consisted of 2561 characters, of which 2026 were constant and 399 were parsimony-informative. A total of 50 isolates were included, of which *P.obsoletum* (Romagn.) Matheny & Esteve-Rav. and *P.perlatum* (Cooke) Matheny & EsteveRav. (*Rimosae* s.s. subclade B, *fide*Larsson et al. 2009) served as outgroup taxa. The following models were selected by jModelTest2 (AIC): TPM2uf+G (ITS1, -lnL = 2070.5127), TrNef (5.8S, -lnL = 261.9437), TPM1uf+I+G (ITS2, -lnL = 1683.9167), TrN+I+G (nrLSU, -lnL = 4608.2667) and TIM2+G (mtSSU, -lnL = 1758.7165). Finally, dataset #3 consisted of 1383 characters, of which 1091 were constant and 205 were parsimony-informative. A total of 67 isolates were included, again with *N.bohemica*, *N.salicis* and *N.submelinoides* as outgroup taxa. For this single-locus dataset, the TrN+I+G model gave the best-scoring tree (nrLSU, -lnL = 5708.4547).

**Figure 1.**
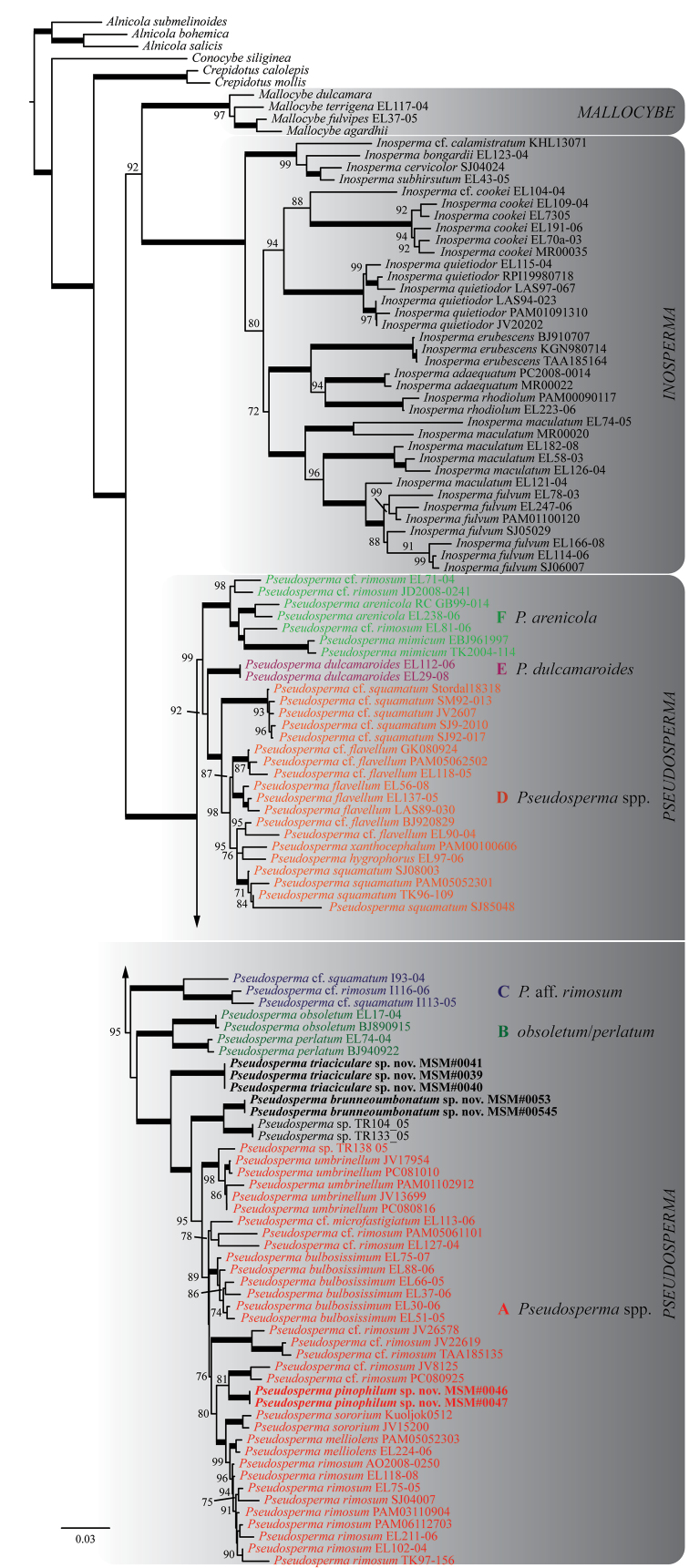
The best-scoring ML tree (-lnL = 27210.474) of the *Rimosae* s.s. and Inosperma clades, reconstructed from the concatenated ITS–nrLSU–mtSSU dataset. ML bootstraps (if ≥ 70) are presented above or in front of the branch leading to each node. Thick branches have maximum support (ML BS = 100). Subclade designations within sect.Rimosae s.s. follow Larsson et al. (2009) in the strict sense. Newly-described species are in boldface.

Six strongly supported clades (referred to as subclades A to F, *fide*Larsson et al. 2009) and two additional clades with maximum support were recovered in the ML analysis of the *Rimosae* s.s. and Inosperma clades (dataset #1, Figure 1). A strongly supported clade with 35 sequences corresponds with *Rimosae* s.s. subclade A and includes the following species: *P.bulbosissimum* (Kühner) Matheny & Esteve-Rav., *P.melliolens* (Kühner) Matheny & Esteve-Rav., *P.pinophilum* sp. nov., *P.rimosum* (Bull.) Matheny & Esteve-Rav. (s.s.), *P.sororium* (Kauffman) Matheny & Esteve-Rav. and *P.umbrinellum* (Bres.) Matheny & Esteve-Rav. In addition, numerous taxa on single branches and less-supported clades are recovered.

**Figure 2.**
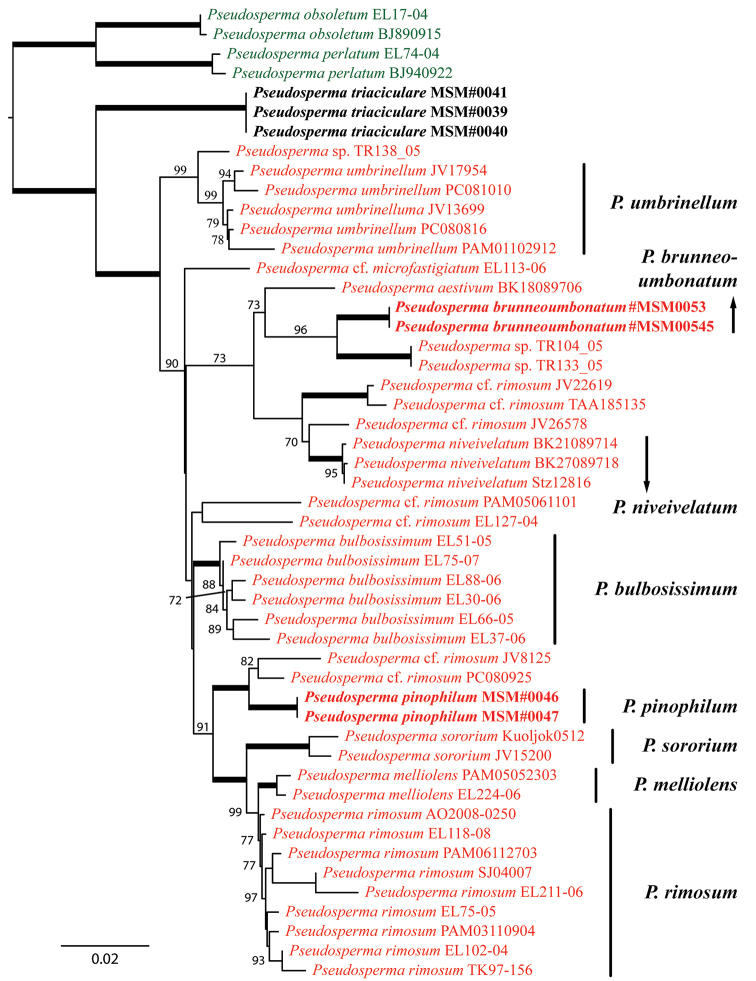
The best-scoring ML tree (-lnL = 9359.879) of *Rimosae* s.s. subclade A, reconstructed from the concatenated ITS–nrLSU–mtSSU dataset. ML bootstraps (if ≥ 70) are presented above or in front of the branch leading to each node. Thick branches have maximum support (ML BS = 100). Well-supported clades that represent described species within *Rimosae* s.s. subclade A are named. Newly-described species are in boldface.

**Figure 3.**
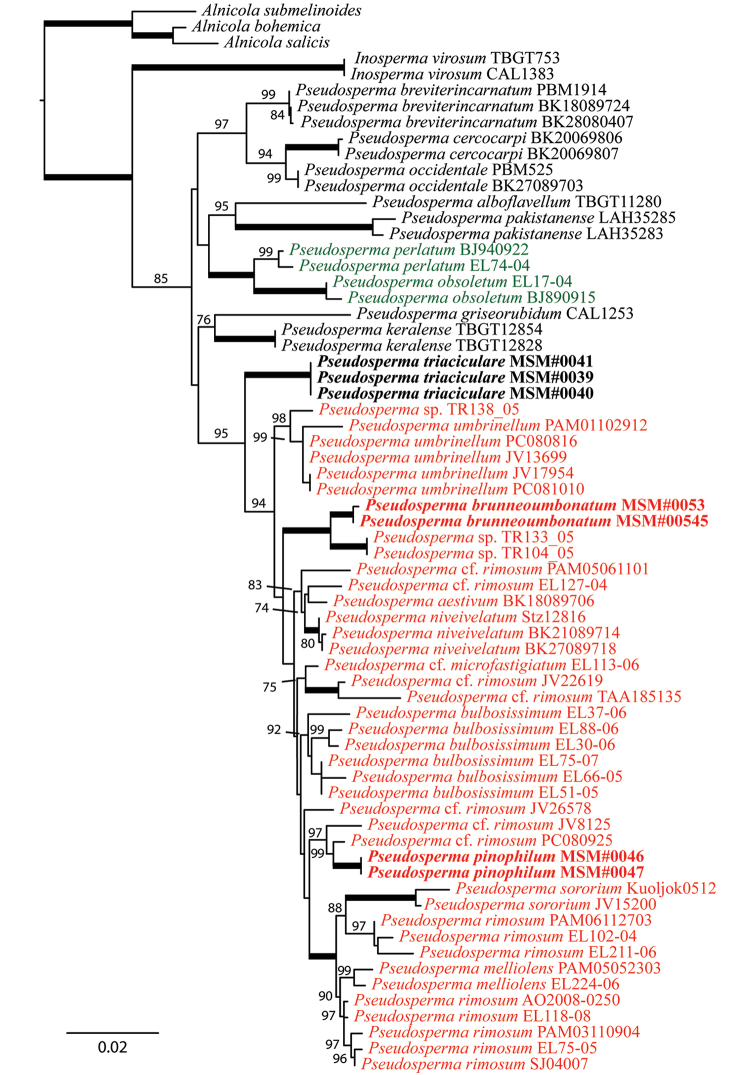
The best-scoring ML tree (-lnL = 5704.951) of *Rimosae* s.s. subclade A, complemented with recently-described species within sect.Rimosae s.s., reconstructed from the nrLSU dataset. ML bootstraps (if ≥ 70) are presented above or in front of the branch leading to each node. Thick branches have maximum support (ML BS = 100). Newly-described species are in boldface.

In all three phylogenetic reconstructions (Figures 1–3), there is high support (BS = 81–100) for the grouping of *P.pinophilum* sp. nov. with P.cf.rimosum from Europe (isolates JV8125 and PC080925). This clade is deeply nested in *Rimosae* s.s. subclade A (*fide*Larsson et al. 2009). *Pseudospermabrunneoumbonatum* sp. nov. is retrieved as sister to an undescribed species from Papua New Guinea (isolates TR104_05 and TR133_05) with high support (BS = 96–100). In both datasets #2 and #3, this clade, again, is deeply nested in *Rimosae* s.s. subclade A. In dataset #1, however, the clade *P.brunneoumbonatum* – *I.* sp. Papua New Guinea is placed between *Rimosae* subclades A and B (*fide*Larsson et al. 2009) with maximum support (Figure 1). *Pseudospermatriaciculare* sp. nov. is retrieved with high support (BS = 95–100) as an independent clade without clear affinities outside of *Rimosae* s.s. subclade A.

Our phylogenetic reconstructions (Figures 1–3) indicate that several undescribed species occur in *Rimosae* s.s. subclade A (see Discussion). All ML analyses recovered two new Pakistani species, *P.triaciculare* and *P.pinophilum*, as strongly-supported lineages nested within this subclade, whereas a third species, *P.brunneoumbonatum*, forms a strongly-supported clade outside of what is currently recognised as subclade A. These three new taxa from Pakistan can be distinguished, based on molecular phylogenetic data, as well as morphology and ecology.

### Taxonomy

#### Pseudosperma brunneoumbonatum

Taxon classificationFungiAgaricalesInocybaceae

Saba & Khalid sp. nov.

4C5835FF-5656-5277-BC03-766EEF3D662C

822655

Figure 4

##### Diagnosis.

Characterised by the dark brown umbo and basidiospores 10.3–15.3(–16.7) × 6.6–9.9 µm and an ecological association with *Pinus*.

**Figure 4.**
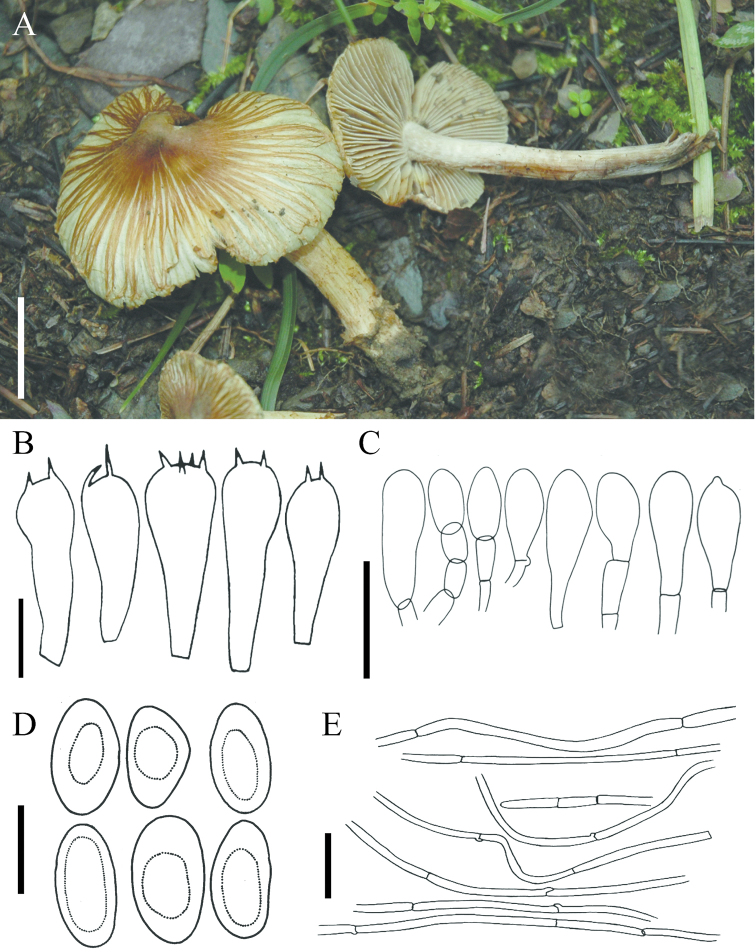
*Pseudospermabrunneoumbonatum*: **A** Basidiomata of holotype collection (LAH 310032) **B–E** microscopic characters: **B** basidia **C** cheilocystidia **D** basidiospores **E** pileipellis. Scale bars: 1 cm (**A**), 10 µm (**B**), 30 µm (**C, E**), 20 µm (**D**).

##### Types.

***Holotype***: Pakistan, Prov. Khyber Pakhtunkhwa, Abbottabad, Shimla, 14 Sep 2012, *leg.* M. Saba & A.N. Khalid; MSM#0053 (LAH 310032); GenBank accession nos. MG742419 (ITS), MG742420 (nrLSU). ***Paratype***: *ibid.*, 6 Aug. 2014; MSM#00545 (LAH 31003); GenBank accession nos. MG742421 (ITS), MG742422 (nrLSU).

##### Etymology.

From Latin, referring to dark brown colour of the umbo.

##### Description.

***Pileus*** 20–38 mm in diam., plane to broadly convex with an acute umbo; margin straight or flaring to deflexed; surface dry, dull, strongly rimose, cracked towards centre but disc smooth and unbroken; strong brown (5YR4/8), disc/umbo deep brown (5YR2/6). ***Lamellae*** regular, adnexed to sinuate, close, pale orange yellow (10YR8/4) or pale yellow (5Y9/4), becoming yellowish-brown with age, concolorous with stipe; edges even; lamelullae one tier; edges white and fimbrirate. ***Stipe*** 22–40 mm, central to slightly eccentric, equal, recurved squamulose, longitudinally fibrillose, pale yellow (5Y9/4) or light yellowish-brown (10YR7/4), veil not observed. Odour spermatic. Context white, lacking any colour changes where cut or bruised.

***Basidiospores*** 10.3–15.3(–16.7) × 6.6–9.9 µm [x = 12.5 × 7.5 µm, Q = 1.2–1.96], smooth, phaseoliform or ellipsoid, thin-walled, pale brown to reddish-brown in KOH, apiculus present or absent, apex obtuse. ***Basidia*** 27–39 × 10.6–16 µm, clavate with refractive contents, primarily 4-sterigmate, less often 2-sterigmate, thin-walled, hyaline in KOH; sterigmata 3–6 µm long. ***Pleurocystidia*** absent. ***Cheilocystidia*** 24–35 × 14–29 µm, numerous, clavate, some catenate, hyaline to pale brown, thin-walled. ***Caulocystidia*** clavate or cylindrical, similar to cheilocystidia, infrequent. ***Pileipellis*** a cutis, hyphae cylindrical, 5–9 µm wide, thin-walled, pale brown in KOH, some with encrustations, septate. ***Lamellar trama*** of parallel hyphae, 5–10 µm wide; subhymenium of compact hyphae, 3–6 µm wide. ***Stipitipellis*** cylindrical hyphae, hyaline in mass in KOH. All structures inamyloid. ***Clamp connections*** present.

##### Habit and habitat.

Occurring in August and September, solitary or in groups, scattered on the forest floor in stands of *Pinusroxburghii* (Pinaceae).

##### Notes.

In all phylogenetic reconstructions (Figures 1–3), *P.brunneoumbonatum* sp. nov. is sister to *Pseudosperma* sp. (isolates TR104_05 and TR133_05). This undescribed species from high-elevations in Papua New Guinea is associated with *Castanopsis* (Fagaceae). Of the north temperate species, *P.brunneoumbonatum* is phylogenetically most closely related to *P.umbrinellum* (Figure 3, Table 2). In terms of morphology, *P.brunneoumbonatum* differs from *P.umbrinellum* by its strong brown pileus with an acute umbo (hazel to cinnamon brown) and somewhat larger basidiospores (measuring 10–13 × 5.5–6.5 μm in *P.umbrinellum*). Other related North American taxa are *P.aestivum* (Kropp, Matheny & Hutchison) Matheny & Esteve-Rav. and *P.niveivelatum* (D.E. Stuntz ex Kropp, Matheny & Hutchison) Matheny & Esteve-Rav. *Pseudospermaaestivum* can be separated by larger basidiomata and different pileus colouration (yellowish to pale yellow with yellow-brown centre), whereas *P.niveivelatum* has a white stipe and a non-rimose pileus with different colouration (covered with abundant white velipellis) (Kropp et al. 2013). *Pseudospermaperlatum* (Cooke) Matheny & Esteve-Rav. superficially resembles *P.brunneoumbonatum*. However, the slightly larger basidiospores, pale orange yellow stipe and a presumed association with *Pinus* distinguish the new species from *P.perlatum*, which is an associate of deciduous trees (Vauras and Huhtinen 1986). It differs from *I.rimosum* in having broader basidiospores.

*Pseudospermaneoumbrinellum* (T. Bau & Y.G. Fan) Matheny & Esteve-Rav. is an Asian species (described from China) with similar basidioma size and colouration (Bau and Fan 2018). The basidiospores of *P.brunneoumbonatum*, however, are remarkably larger. *Pseudospermahimalayense* (Razaq, Khalid & Kobayashi) Matheny & Esteve-Rav. was recently described from Pakistan (Liu et al. 2018) and is similar to *P.brunneoumbonatum* in having similar pileus size. This species was found at different localities in the western Himalayas, but always near *Pinuswallichiana*. *Pseudospermahimalayense* has a much longer stipe (50–80 mm vs. max. 40 mm in *P.brunneoumbonatum*); white to pale yellow, olive yellow or light brown pileus; and somewhat smaller basidiospores. *Pseudospermapakistanense* (Z. Ullah, S. Jabeen, H. Ahmad & A.N. Khalid) Matheny & Esteve-Rav., another species described from Pakistan, can be differentiated by the presence of pleurocystidia, somewhat smaller basidiospores and phylogenetic placement (Ullah et al. 2018, Figure 3).

The following two species have not yet been recombined in *Pseudosperma*. However, phylogenetic evidence undoubtedly places both *I.neglecta* E. Horak, Matheny & Desjardin and *I.friabilis* Matheny & Kudzma in the newly-recognised genus *Pseudosperma* (Horak et al. 2015, Matheny and Kudzma 2019). The new combinations are presented at the end of the taxonomy section. *Inocybeneglecta* from Thailand was described in the Pseudosperma clade by Horak et al. (2015). While it also lacks pleurocystidia and has a strong brown umbonate pileus, it is different from *P.brunneoumbonatum* by the smaller pileus (12–18 mm vs. 20–38 mm) and smaller and differently-shaped basidiospores. In addition, *I.neglecta* is only known from the type locality, growing in a tropical montane forest dominated by *Lithocarpus* Blume and *Castanopsis* (D. Don) Spach (both in Fagaceae). *Inocybefriabilis*, described from North America in the Pseudosperma clade, resembles *P.brunneoumbonatum* by lacking pleurocystidia and having a similarly coloured pileus. However, *I.friabilis* has smaller basidiospores, is associated with *Quercus* and *Carya* and has an eastern United States distribution.

In *The taxonomic studies of the genus Inocybe*, Kobayashi (2002) discussed 136 species, of which 13 (including four varieties and three formae) in subgenus InospermasectionRimosae. These are [all referred to as *Inocybe* in Kobayashi (2002)]: *Inospermaadaequatum* (Britzelm.) Matheny & Esteve-Rav., *I.aureostipes* (Kobayasi) Matheny & Esteve-Rav., *I.cookei* (Bres.) Matheny & Esteve-Rav., *I.erubescens* (A. Blytt) Matheny & Esteve-Rav. [as its synonym *I.patouillardii* Bres.], *I.maculatum* (Boud.) Matheny & Esteve-Rav., *Pseudospermaavellaneum* (Kobayasi) Matheny & Esteve-Rav., *P.bisporum* (Hongo) Matheny & Esteve-Rav., *P.flavellum* (P. Karst.) Matheny & Esteve-Rav., *P.macrospermum* (Hongo) Matheny & Esteve-Rav., *P.rimosum* [as its synonym *Inocybefastigiata* (Schaeff.) Quél.], *P.squamatum* (J.E. Lange) Matheny & Esteve-Rav., *P.transiens* (Takah. Kobay.) Matheny & Esteve-Rav. and *P.umbrinellum*. Since no sequence data are available for *P.avellaneum*, *P.bisporum*, *P.macrospermum* and *P.transiens*, we will compare their morphology with the newly-proposed Pakistani species.

*Pseudospermaavellaneum* has a pale greyish ochraceous pileus, its basidiospores are smaller and its cheilocystidia are distinctly narrower (width 9.5–14.5 vs. 14–29 μm) compared to *P.brunneoumbonatum*. As the only species in sect.Rimosae (*sensu*Kobayashi 2002), *P.bisporum* is 2-sterigmate. In addition, this species has a generally shorter stipe (17–26 vs. 22–40 mm in *P.brunneoumbonatum*), the edges of its lamellae are serrate (with small teeth as a saw) and, again, the cheilocystidia are narrower (width 10.0–13.8 vs. 14–29 μm in *P.brunneoumbonatum*). Another Japanese species, *P.macrospermum*, is morphologically different in the following characters: the stipe has a bulbous base, the basidia are shorter and narrower and its pileus is much smaller in diameter. Finally, *P.transiens* has a much longer stipe, its basidia are always narrower (up to 9.5 μm wide) and its cheilocystidia are both longer and narrower ((29–)38–52 × 9.5–13.8 μm) compared to *P.brunneoumbonatum*.

**Table 2.** Comparison of ecological and morphological characters among the three newly described Pakistani species of *Pseudosperma* and phylogenetically similar species *P.rimosum* and *P.umbrinellum*.

Species	*P.brunneoumbonatum*	*P.pinophilum*	*P.triacicularis*	*P.rimosum*	*P.umbrinellum*
**Host association(s)**	*Pinus*	*Pinus*	*Pinus*	*Abies*, *Alnus*, *Betula*, *Carpinus*, *Cedrus*, *Corylus*, *Fagus*, *Larix*, *Picea*, *Pinus*, *Populus*, *Quercus*, *Salix*, *Tilia*	*Helianthemum*, *Pinus*, *Populus*, *Quercus*
**Pileus color**	Strong brown (5YR4/8), disc/umbo deep brown (5YR2/6)	Strong brown throughout (5YR4/6 to 5YR4/8), with dark brown umbo	Brownish orange (5YR5/8) to fulvous	Highly variable, from pale to ochraceous yellow brown to dark brown, usually darkest around center; sometimes very conspicuous and bright yellow; sometimes blackish brown	Hazel to cinnamon brown, warm yellowish to reddish brown caps with a dark center and contrasting strongly rimose and lighter periphery
**Umbo**	Acute	Acute	Acute to subacute or obtuse	Acute	Blunt
**Velipellis**	Absent	Absent	Present	Absent	Absent
**Basidiospores**	10.3–15.3(–16.7) × 6.6–9.9 µm	(8.2–)9.4–15.8 × 6.3–8 µm	(7.7–)8.9–12.5 × 6.1–7.7 µm	9.5–12.5 × 6.0–7.0 µm	10.0–13.0 × 5.5–6.5 µm
**Reference(s)**	This paper	This paper	This paper	Kuyper (1986), Larsson et al. (2009)	Kuyper (1986), Larsson et al. (2009)

#### Pseudosperma pinophilum

Taxon classificationFungiAgaricalesInocybaceae

Saba & Khalid sp. nov.

4EE9A18C-5321-5693-9289-1D4700A8B84E

822656

Figure 5

##### Diagnosis.

Characterised by the pale to light yellow equal stipe, basidiospores (8.2–)9.4–15.8 × 6.3–8 µm and an ecological association with *Pinus*.

**Figure 5.**
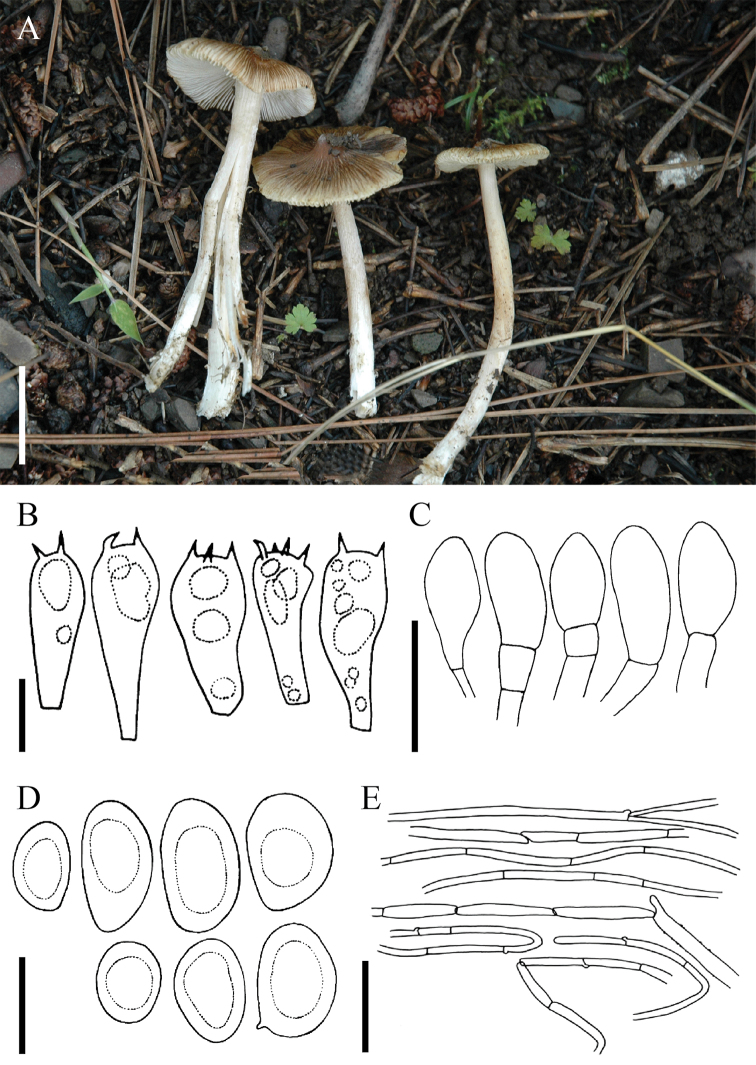
*Pseudospermapinophilum*: **A** Basidiomata of holotype collection (FH 00304582) **B–E** microscopic characters: **B** basidia **C** cheilocystidia **D** basidiospores **E** pileipellis. Scale bars: 1 cm (**A**), 10 µm (**B, D**), 30 µm (**C, E**).

##### Types.

***Holotype***: Pakistan, Prov. Khyber Pakhtunkhwa, Abbottabad, Shimla, 14 Sep 2012, *leg.* M. Saba & A.N. Khalid; MSM#0046 (FH 00304582); GenBank accession nos. MG742414 (ITS), MG742418 (nrLSU), MG742416 (mtSSU). ***Paratype***: Pakistan, Prov. Khyber Pakhtunkhwa, Shangla, Yakh Tangay, under *Pinuswallichiana*, 2 Sep 2013, *leg.* M. Saba & A.N. Khalid; MSM#0047 (LAH 310049); GenBank accession nos. MG742417 (ITS), MG742415 (nrLSU), MK474612 (mtSSU).

##### Etymology.

From Greek, referring to an association with pine species.

##### Description.

***Pileus*** 16–31 mm in diam., convex, broadly convex or plane with an acute umbo; margin straight or flaring to deflexed; surface dry, dull, rimose, cracked towards centre, strong brown throughout (5YR4/6 to 5YR4/8) with dark brown umbo. ***Lamellae*** regular, adnexed to sinuate, close, white when young, light olivaceous at maturity; edges even. ***Stipe*** 54–70 mm, central, equal, longitudinally fibrillose, white with pale greenish-yellow (10Y9/4) or light yellow (5Y9/6) tinge or olivaceous tinge; veil not observed. Context white. Odour not distinctive.

***Basidiospores*** (8.2–)9.4–15.8 × 6.3–8.0 µm [x = 13.5 × 7.6 µm, Q = 1.4–1.9], smooth, phaseoliform or ellipsoid, thin-walled, pale brown to golden brown in KOH, apiculus small and not distinctive, apex obutse. ***Basidia*** 21–40 × (9–)11–14 µm, clavate with refractive contents, primarily 4-sterigmate, less often 2-sterigmate, thin-walled, hyaline in KOH; sterigmata 2.5–4.0 µm long. ***Pleurocystidia*** absent. ***Cheilocystidia*** 25–47 × 10–20 µm, numerous, clavate or cylindrical, hyaline to pale brown in KOH, thin-walled. ***Caulocystidia*** not observed. ***Pileipellis*** a cutis of repent hyphae, hyphae cylindrical, 4–12 µm wide, thin-walled, pale brown in KOH, septate. ***Lamellar trama*** of parallel hyphae, 5–11 µm wide; subhymenium of compact hyphae, 3–6 µm wide. ***Stipitipellis*** cylindrical hyphae, 5–12 µm wide, hyaline in mass in KOH; all structures inamyloid. ***Clamp connections*** present.

##### Habit and habitat.

Occurring in September, solitary or in groups, scattered on the forest floor in stands of *Pinusroxburghii* and *P.wallichiana* (Pinaceae).

##### Notes.

Both *P.brunneoumbonatum* and *P.pinophilum* are placed in sect.Rimosae s.s. subclade A (Figures 1–3), which corresponds to *P.rimosum* senso lato, including the several *formae* and variations described for this species (Larsson et al. 2009). *Pseudospermapinophilum* clusters with P.cf.rimosum (isolates JV1825 and PC080925). The pale yellow to light yellow tinged, equal stipe in *P.pinophilum* is very different compared to the white (rarely tinged with ochre), sub-bulbous stipe typical for *P.rimosum*. Moreover, *P.pinophilum* has broader basidiospores ((8.2–)9.4–15.8 × 6.3–8.0 µm) compared to *P.rimosum* (9–11(–13) × 4.5–6.0 µm). Also *P.brunneoumbonatum* has broader – and generally larger – basidiospores (10.3–15.3(–16.7) × 6.6–9.9 µm) compared to *P.rimosum*. *Pseudospermasororium* is relatively closely related to *P.pinophilum* and can be differentiated in having different pileus colouration (greyish-brown to pinkish-grey or pale pinkish-beige) and measurement of basidiospores (10–12.5 × 5.5–6.0 µm) (Kauffman 1926).

Two more species of *Pseudosperma* are known from Pakistan; both *P.himalayense* and *P.pakistanense* were described, based on material collected in Pakistan. *Pseudospermahimalayense* was found near *Pinuswallichiana* trees, but an ITS sequence generated from root tips (GenBank acc. no. HG796995) confirmed an ectomycorrhizal association with *Quercusincana* (Liu et al. 2018). It can be distinguished from *P.pinophilum* by the pale yellowish to camel brown, fibrillose pileus; longer cheilocystidia (43–60 µm vs. 25–47 µm); and much thicker pileipellis. In addition, *P.himalayense* was resolved as sister to P.cf.microfastigiatum (Kühner) Matheny & Esteve-Rav. in Liu et al.’s (2018)ITS phylogeny. *Pseudospermapakistanense* was found in a mixed conifer-dominated forest with some deciduous trees, under *Quercusincana* (Ullah et al. 2018). This species can be differentiated from the new species by the presence of pleurocystidia, the smaller stipe (50 mm vs. 54–70 mm in *P.pinophilum*) and its phylogenetic position (Ullah et al. 2018). In our nrLSU phylogeny, *P.pakistanense* was retrieved as sister to *P.alboflavellum* (C.K. Pradeep & Matheny) Haelew. (Figure 3).

The Japanese species in sect.Rimosae without sequence data from Kobayashi (2002), *P.avellaneum*, *P.bisporum*, *P.macrospermum* and *P.transiens*, are also different from *P.pinophilum* in their morphology. *Pseudospermaavellaneum* has smaller basidiospores and the pileipellis hyphae are almost hyaline (vs. pale brown in *P.pinophilum*). *Pseudospermabisporum* has lamellae with serrate edges, its stipe is much shorter (17–26 vs. 54–70 mm in *P.pinophilum*), the basidia are 2-sterigmate, the cheilocystidia are usually shorter (max. 31 µm in length) and the pileipellis hyphae are smaller in diameter. *Pseudospermamacrospermum* has a smaller pileus diameter, a shorter stipe, narrower basidia, usually shorter cheilocystidia and pileipellis hyphae that are smaller in diameter. Finally, both the basidiospores (4.8–6.5 vs. 6.3–8.0 µm in *P.pinophilum*) and basidia (8.8–9.5 vs. (9–)11–14 µm in *P.pinophilum*) of *P.transiens* are narrower. In addition, the cheilocystidia of *P.pinophilum* are hyaline to pale brown in KOH, whereas in *P.transiens*, they are “rarely filled with yellowish brown contents” (Kobayashi 2002).

#### Pseudosperma triaciculare

Taxon classificationFungiAgaricalesInocybaceae

Saba & Khalid sp. nov.

A06477AF-CF89-58AF-89B3-11E1537BE971

822657

Figure 6

##### Diagnosis.

Characterised by the acutely umbonate brownish-orange to fulvous pileus, the presence of a pale velipellis coating on the pileus, septate cheilocystidia and an ecological association with *Pinus*.

**Figure 6.**
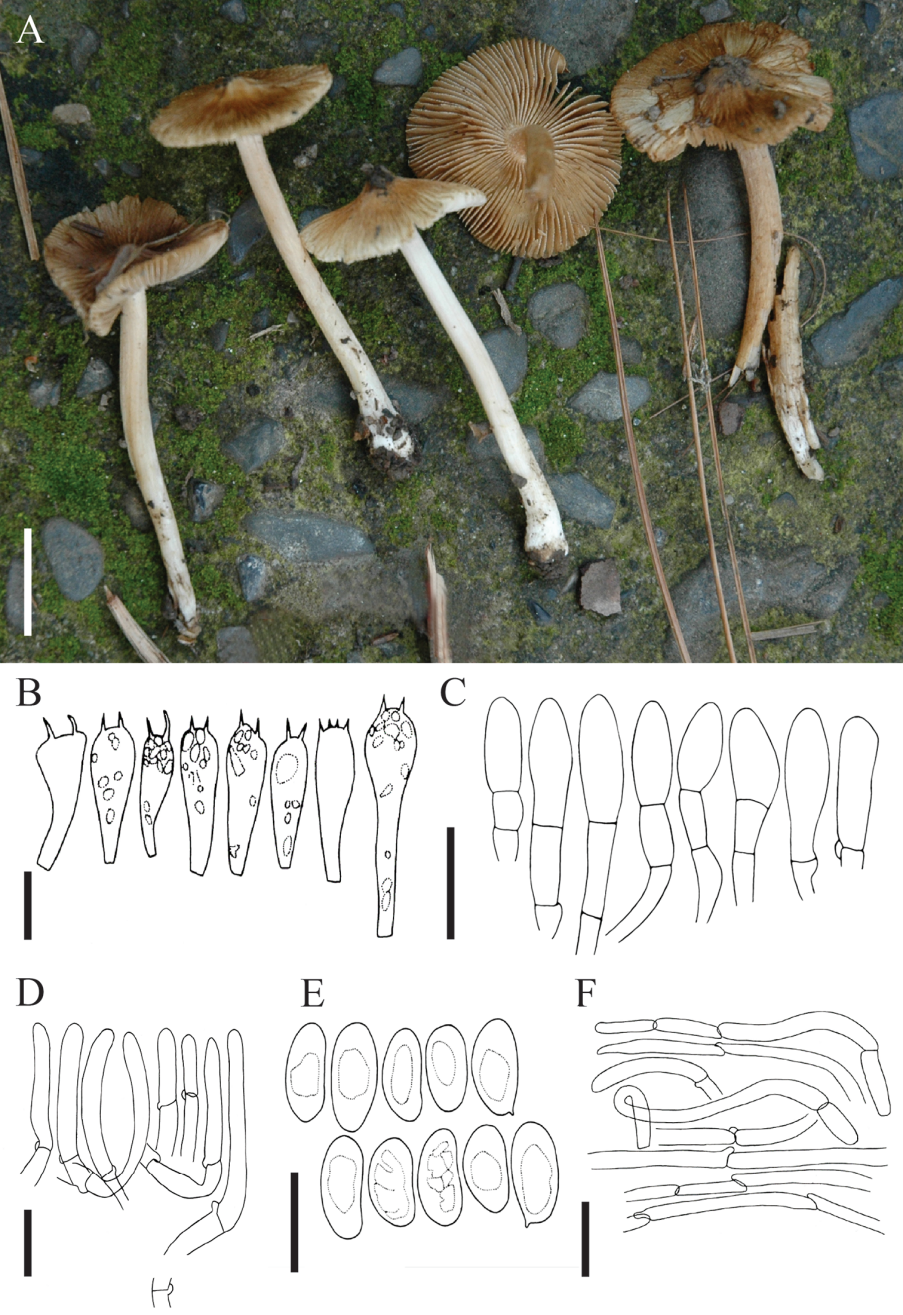
*Pseudospermatriaciculare*: **A** Basidiomata of paratype collection (FH 00304561) **B–F** microscopic characters: **B** Basidia **C** cheilocystidia **D** caulocystidia **E** basidiospores **F** pileipellis. Scale bars: 1 cm (**A**), 10 µm (**B, E**), 30 µm (**C, D, F**).

##### Types.

***Holotype***: Pakistan, Prov. Khyber Pakhtunkhwa, Mansehra, Batrasi, under *Pinusroxburghii*, 3 Aug 2014, *leg.* M. Saba & A.N. Khalid; MSM#0039 (LAH 310054); GenBank accession nos. MG742423 (ITS), MG742424 (nrLSU), MG742425 (mtSSU). ***Paratypes***: *ibid.*, 3 Aug 2014; MSM#0040 (LAH 310055); GenBank accession nos. MG742426 (ITS), MG742427 (nrLSU), MG742428 (mtSSU). *Ibid.*, 3 Aug 2014; MSM#0041 (LAH 310056); GenBank accession nos. MG742429 (ITS), MG742430 (nrLSU), MG742431 (mtSSU). Pakistan, Prov. Khyber Pakhtunkhwa, Abbottabad, Shimla, 14 Sep 2012, *leg.* M. Saba & A.N. Khalid; MSM#0038 (FH 00304561).

##### Etymology.

From Latin, meaning “three-needled,” with reference to the association with the three-needled pine *Pinusroxburghii*.

##### Description.

***Pileus*** 12–29 mm in diam., conical when young, plane to convex at maturity, with acute to subacute or obtuse umbo; margin radially rimose, straight or flaring to uplifted; surface dry, dull, colour brownish-orange (5YR5/8) to fulvous, presence of a pale velipellis coating over the disc. ***Lamellae*** regular, adnexed to sinuate, close, pale orange yellow (10YR8/4), edges even; two tiers of lamelullae. ***Stipe*** 19–60 mm, central, equal, fibrillose, white with pale orange yellow tinge (10YR8/4). Odour mild, not diagnostic.

***Basidiospores*** (7.7–)8.9–12.5 × 6.1–7.7 µm [x = 10.2 × 6.9 µm, Q = 1.64–2.2], smooth, mostly elliptic, thin-walled, yellowish-brown in KOH, apiculus present small and indistinctive. ***Basidia*** 24–36 × (9–)10–13 µm, clavate to broadly clavate with refractive contents, 4-sterigmate, thin-walled, hyaline in KOH; sterigmata 2.5–4.0 µm long. ***Pleurocystidia*** absent. ***Cheilocystidia*** cylindrical to clavate, septate, some with sub-capitate apices, terminal cells 23–54 × 9–16 µm, non-encrusted, hyaline, thin-walled. ***Caulocystidia*** 36–98 × 7–14 µm, cylindrical, non-encrusted, hyphoid, thin-walled. ***Pileipellis*** a cutis, hyphae cylindrical, 6–12 µm wide, thin-walled, golden brown or yellowish-brown in KOH, without encrustations, septate. ***Lamellar trama*** of parallel hyphae, 6–12 µm wide; subhymenium of compact hyphae, 3–6 µm wide. ***Stipitipellis*** cylindrical hyphae, 2–12 µm wide, hyaline in mass in KOH; all structures inamyloid. ***Clamp connections*** present.

##### Habit and habitat.

Occurring in August to September, solitary or in groups, scattered on the forest floor in stands of *Pinusroxburghii* (Pinaceae).

##### Notes.

*Pseudospermatriaciculare* has been found in association with *Pinusroxburghii*, the three-needled pine. This new species forms a distinct monophyletic group without clear affinities outside of *Rimosae* s.s. subclade A (Figures 1–3). Some of the unique features of this species are the umbonate brownish-orange to pale orange yellow pileus; cylindrical to clavate cheilocystidia; and cylindrical, non-encrusted, hyphoid caulocystidia. Allied species include *P.brunneoumbonatum*, *P.griseorubidum* (K.P.D. Latha & Manim.) Matheny & Esteve-Rav., *P.keralense* [synonym *I.rimulosa* C.K. Pradeep & Matheny] and *P.umbrinellum*. *Pseudospermatriaciculare* shares the same presumed *Pinus* association and shape of basidiomata with *P.brunneoumbonatum*, but can be distinguished by its brownish-orange pileus and smaller basidiospores. *Pseudospermaumbrinellum* is differentiated from *P.triaciculare* by the presence of an obtuse umbo (acute in *P.triaciculare*), yellowish- or reddish-brown pileus (brownish-orange in *P.triaciculare*), somewhat narrower basidiospores (5.5–6.5 µm vs. 6.1–7.7 µm) and a broad host range, including species in Cistaceae, Fagaceae, Pinaceae and Salicaceae (Larsson et al. 2009).

*Pseudospermatriaciculare* is most closely related to *P.griseorubidum* and *P.keralense*, described recently from tropical India (Latha and Manimohan 2015, Pradeep et al. 2016, Figure 3). *Pseudospermagriseorubidum* can be differentiated by its pileus, which is greyish-red and rarely with an umbo. In addition, *P.griseorubidum* is associated with members of Dipterocarpaceae (Latha and Manimohan 2015). The differences between *P.keralense* and *P.triaciculare* are more subtle. *Pseudospermakeralense* can be separated based on the following features: its lamellae have serrate edges and its basidiospores are narrower on average (6.1 vs. 6.9 µm in *P.triaciculare*). It is also phylogenetically clearly different; the ITS sequence of the holotype collection (GenBank acc. no. KM924523) is 84.11% identical to the holotype of *P.triaciculare*, whereas the LSU (KM924518) is 95.13% identical.

Other similar Asian species include *P.himalayense*, *P.neoumbrinellum*, *P.pakistanense* and *P.yunnanense* (T. Bau & Y.G. Fan) Matheny & Esteve-Rav. *Pseudospermatriaciculare* resembles *P.neoumbrinellum* in its pileus and basidiospores. However, it is easily differentiated by the characteristic brownish-orange to fulvous colouration of its pileus, whereas the pileus of *P.neoumbrinellum* is chocolate to dark brown in colour (Bau and Fan 2018). In addition, the shape and size of caulocystidia in these two species are very different: 20–48 × 10–17 µm in *P.neoumbrinellum* vs. 36–98 × 7–14 µm in *P.triaciculare*. *Pseudospermatriaciculare* is different from the recently-described *P.himalayense* from Pakistan (Liu et al. 2018) by the presence of a velipellis and a shorter stipe (16–60 vs. 50–80 µm). *Pseudospermapakistanense* is separated from *P.triaciculare* by the absence of velipellar hyphae (unless the authors referred to the velipellis by their description of “[pileus] sometimes peeling off in the form of fine threads”), presence of pleurocystidia and a generally wider stipitipellis lacking caulocystidia (Ullah et al. 2018). Finally, *P.yunnanense*, described from China, also has velipellar hyphae, but its basidiomata are much larger in size (pileus 30–60 mm in diam., stipe 60–70 mm) and it lacks caulocystidia (Bau and Fan 2018). We did not include *P.yunnanense* in our phylogenetic analyses, but blasted the ITS sequence of the holotype collection (GenBank acc. no. MH047250) against *P.triaciculare*, resulting in 89.09% identity. *Pseudospermayunnanense* is phylogenetically most similar to *P.perlatum*.

Finally, *P.avellaneum*, *P.bisporum*, *P.macrospermum* and *P.transiens* from Kobayashi’s (2002) morphological *Inocybe* treatment are all different from *P.triaciculare*. Of all four, *P.avellaneum* is probably most difficult to separate from the new species: its pileus is pale greyish-ochraceous, the stipe is less slender and – this seems the best character for separating both species – no caulocystidia were observed. *Pseudospermabisporum* has lamellae with serrate edges, 2-sterigmate basidia and pileipellis hyphae that are smaller in diameter. In addition, again, no caulocystidia were observed in this species. Compared to *P.triaciculare*, the basidiospores of *P.macrospermum* are longer (10.5–)14.0–15.5(–18.3) vs. (7.7–)8.9–12.5) µm, its basidia are narrower (8.8–9.5(–12.5) vs. (9–)10–13 µm) and its cheilocystidia are wider (16–18 vs. 9–16 µm). *Pseudospermatransiens* has basidiospores (4.8–6.5 vs. 6.1–7.7 µm) and basidia (8.8–9.5 vs. (9–)10–13 µm) that are both narrower than those in *P.triaciculare*. In addition, the pileus of *P.transiens* is coloured brown to dark brown, whereas *P.triaciculare* has a brownish-orange to fulvous pileus.

### New combinations

During our studies of *Inocybe* sensu lato, we came across species of *Inocybe* that had not been recombined in the appropriate genera after Matheny et al. (2019) proposed a new generic system. Five names are recombined in *Inosperma*, *Mallocybe* and *Pseudosperma*.

#### Inosperma vinaceobrunneum

Taxon classificationFungiAgaricalesInocybaceae

(Matheny, Ovrebo & Kudzma) Haelew., Index Fungorum 436: 1 (2020).

69B3C893-DC3D-5EA9-990A-AE4D321FDCE7

Index Fungorum No: IF557431

≡ Inocybevinaceobrunnea Matheny, Matheny and Kudzma, J. Torrey Bot. Soc. 146(3): 227 (2019). [Basionym]

##### Note.

This combination was made, based on a four-locus phylogeny (ITS, nrLSU, rpb1, rpb2). *Inospermavinaceobrunneum* was retrieved in a clade with two other species (*I.rodiolum* (Bres.) Matheny & Esteve-Rav. and an undescribed species), sister to *I.adaequatum* (Matheny and Kudzma 2019).

#### Mallocybe erratum

Taxon classificationFungiAgaricalesInocybaceae

(E. Horak, Matheny & Desjardin) Haelew. comb. nov.

1DBFCB28-3E90-5583-843A-990C080D2EFA

Index Fungorum No: IF557512

≡ Inocybeerrata E. Horak, Matheny & Desjardin, Phytotaxa 230(3): 210 (2015). [Basionym]

##### Note.

This combination is based on phylogenetic evidence of the holotype (Horak et al. 2015). Based on both nrLSU-alone and nrLSU–rpb1–rpb2 datasets, it is placed deep in *Mallocybe*. It is highly supported as a sister species to an undescribed Zambia species (“*I.microdulcamara*” nom. prov.), both sister to *M.heimii* (Bon) Matheny & Esteve-Rav. (Matheny et al. 2009, Horak et al. 2015).

#### Pseudosperma alboflavellum

Taxon classificationFungiAgaricalesInocybaceae

(C.K. Pradeep & Matheny) Haelew., Index Fungorum 436: 1 (2020).

6CE9ECF9-3D03-50BB-9282-2893F6D40FD0

Index Fungorum No: IF557432

≡ Inocybealboflavella C.K. Pradeep & Matheny, Pradeep et al., Mycol. Progr. 15: 13 (2016). [Basionym]

##### Note.

This combination was made, based on phylogenetic placement of the isotype (Pradeep et al. 2016, this study). In our nrLSU phylogeny, it was retrieved as a sister species to *P.pakistanense* with high support (Figure 3).

#### Pseudosperma friabile

Taxon classificationFungiAgaricalesInocybaceae

(Matheny & Kudzma) Haelew., Index Fungorum 436: 1 (2020).

2F55F2A2-5DA7-5107-BB66-B0385AE9B781

Index Fungorum No: IF557433

≡ Inocybefriabilis Matheny & Kudzma, J. Torrey Bot. Soc. 146(3): 226 (2019). [Basionym]

##### Note.

This combination was made, based on phylogenetic evidence. *Pseudospermafriabile* is most closely related to *P.gracilissimum* (Matheny & Bougher) Matheny & Esteve-Rav. and *P.keralense* (K.P.D. Latha & Manim.) Matheny & Esteve-Rav., deep in the Pseudosperma clade (*fide*Matheny 2005, Matheny and Kudzma 2019).

#### Pseudosperma neglectum

Taxon classificationFungiAgaricalesInocybaceae

(E. Horak, Matheny & Desjardin) Haelew. comb. nov.

9D7286CA-1A37-57DC-9F0C-3DEC35B729F3

Index Fungorum No: IF557513

≡ Inocybeneglecta E. Horak, Matheny & Desjardin, Phytotaxa 230(3): 208 (2015). [Basionym]

##### Note.

The combination of *I.neglecta* in genus *Pseudosperma* is made, based on phylogenetic evidence. Horak et al. (2015) presented the phylogenetic reconstruction of an nrLSU dataset and found high statistical support for the Pseudosperma clade (*fide*Matheny 2005) including *P.neglectum*. While *P.neglectum* was retrieved as sister to the remaining members of the Pseudosperma clade, there was no support for this relationship. The same result was also found by Kropp et al. (2013). In addition, blasting the ITS sequence of the holotype (GenBank acc. no. EU600829) against sequences from type materials, resulted in *P.occidentale* (Kropp, Matheny & Hutchison) Matheny & Esteve-Rav. and *P.illudens* (Matheny, Bougher & G.M. Gates) Matheny & Esteve-Rav. with the highest percentages of identity (96.46% and 96.28%, respectively).

## Discussion

Pakistan is located in southern Asia. This country is geographically diverse, ranging from the mountainous northern part, where the Himalayas meet their westernmost end, to the southern part with the coastal area along the Arabian Sea. Following the Köppen-Geiger classification system for climate, 20 types can be found in Pakistan – including four arid, six temperate, eight cold and even two polar (Beck et al. 2018). Note that despite this diversity in climate types, most of the country has a hot desert climate (*BWh*, Peel et al. 2007). Pakistan has a very rich flora; in an ongoing effort to write the *Flora of Pakistan*, S.I. Ali and colleagues identified 5,521 plant species in 1,572 genera thus far (Ali 2008). When keeping the ratio between vascular plants and fungi (1:6) in mind (*sensu*Hawksworth 1991), this number of plants only hints at the true potential of in-depth mycological studies in Pakistan, which has been traditionally under-explored.

The multiple geographic features, different climates and plant species richness in Pakistan are suggestive of a high diversity of fungal species. In recent years, many papers have been published, describing new species from different fungal groups collected in Pakistan (e.g. Razaq et al. 2012, Nawaz et al. 2013, Thongklang et al. 2014, Qasim et al. 2015a, 2015b, Sarwar et al. 2015, Hussain et al. 2016, 2017, 2018, Jabeen et al. 2016, Farooqi et al. 2017, Naseer et al. 2018, Ullah et al. 2018, Saba et al. 2019a, 2019b, Kiran et al. 2020). Thirty-five species of *Inocybe* sensu lato are reported from Pakistan (Ahmad et al. 1997, Ilyas et al. 2013, Saba et al. 2015, Jabeen et al. 2016, Farooqi et al. 2017, Razaq and Shahzad 2017, Naseer et al. 2018, Ullah et al. 2018, Song et al. 2019, this study). The genus *Pseudosperma* is poorly known in Pakistan, with only three species that were known before this study: *P.himalayense*, *P.rimosum* and *P.pakistanense* (Ahmad et al. 1997, Liu et al. 2018, Ullah et al. 2018).

In his dissertation about smooth-spored species of *Inocybe* from Europe, Kuyper (1986) presented a key to species of sect.Rimosae. He included 12 species [all as *Inocybe*]: *Inospermaadaequatum*, *I.cookei*, *I.erubescens*, *I.maculatum*, *I.quietiodor* (Bon) Matheny & Esteve-Rav., *I.reisneri* (Velen.) Matheny & Esteve-Rav., *Pseudospermaarenicola* (R. Heim) Matheny & Esteve-Rav., *P.flavellum*, *P.mimicum* (Massee) Matheny & Esteve-Rav., *P.rimosum* (sensu lato), *P.squamatum* and *I.vinosistipitatum* (Grund & D.E. Stuntz) Matheny & Esteve-Rav. Kuyper (1986) followed a conservative approach for *P.rimosum* – citing 31 species and varieties as synonyms and allowing considerable morphological plasticity and broad ecological amplitude. Larsson et al. (2009) followed a less conservative approach and recognised *P.obsoletum*, *P.perlatum* and *P.umbrinellum* as separate species in their identification key of Maculata and *Rimosae* s.s. clades in north-western Europe. These three species were amongst the synonymies of *P.rimosum* as treated by Kuyper (1986). Following both keys, our newly described taxa are most similar to *P.rimosum* and *P.umbrinellum* (Table 2). From our phylogenetic analyses, it is obvious that both *P.rimosum* and *P.umbrinellum* are separated from our Pakistani species. Other, more recently described taxa of *Pseudosperma* are also differentiated from the newly-proposed species, based on morphology, molecular phylogeny and geographic distribution.

Our phylogenetic analyses revealed that several undescribed species or collections that have not yet been properly identified occur in *Rimosae* s.s. subclade A (Larsson et al. 2009, Kropp et al. 2012). These are represented by singleton clades and clades including tentatively (cf.) or unidentified isolates. For example, isolates TR104_05 and TR133_05 represent an undescribed species from Papua New Guinea. In addition, isolates JV1825, PC080925, JV22619 and TAA185135 were identified as P.cf.rimosum, but represent at least two different species, either undescribed or previously described, but without available DNA sequence data. The isolate JV26578, which forms a singleton clade with unresolved position in our phylogenetic analyses, was also identified as P.cf.rimosum, but this identification is again inaccurate. We agree with Larsson et al. (2009) that more taxa need be sampled before the diversity and evolutionary relationships in this section can be fully understood.

## Data availability

All holotype and paratype collections of the new species are deposited at LAH and FH. The sequences generated during this study are deposited in NCBI GenBank under accession numbers MG742414–MG742431. The sequence alignments generated in the present study are available from figshare (https://doi.org/10.6084/m9.figshare.c.4701338).

## Supplementary Material

XML Treatment for Pseudosperma brunneoumbonatum

XML Treatment for Pseudosperma pinophilum

XML Treatment for Pseudosperma triaciculare

XML Treatment for Inosperma vinaceobrunneum

XML Treatment for Mallocybe erratum

XML Treatment for Pseudosperma alboflavellum

XML Treatment for Pseudosperma friabile

XML Treatment for Pseudosperma neglectum
